# Systematic investigation of global coordination among mRNA and protein in cellular society

**DOI:** 10.1186/1471-2164-11-364

**Published:** 2010-06-09

**Authors:** Haiyun Wang, Qi Wang, Utz J Pape, Bairong Shen, Jianhua Huang, Bin Wu, Xia Li

**Affiliations:** 1 School of Life Science and Technology, Tongji University, Shanghai 200092, China; 2 Huashan Hospital, Fudan University, Shanghai 200040, China; 3 Department of Biostatistics and Computational Biology, Dana-Farber Cancer Institute and Harvard School of Public Health, Boston, MA 02115, USA; 4 Department of Pathology, Immune Disease Institute and Harvard Medical School, Boston, MA 02115, USA; 5 Center for Systems Biology, Soochow University, Suzhou 215006, China; 6 College of Bioinformatics Science and Technology, Harbin Medical University, Harbin 150086, China; 7 Institute of Medical Technology, University of Tampere, Tampere 33014, Finland

## Abstract

**Background:**

Cell functions depend on molecules organized in the cellular society. Two basic components are mRNA molecules and proteins. The interactions within and between those two components are crucial for carrying out sophisticated cell functions. The interplay can be analyzed by comparing expression levels of mRNA and proteins. This is critical for understanding the molecular interactions, (post-) transcriptional regulations and conservation of co-expression between mRNAs and proteins. By using high-throughput transcriptome and proteome data, this study aims to systematically investigate the general picture of such expression correlations. We analyze four groups of correlations: (i) transcript levels of different genes, (ii) protein levels of different genes, (iii) mRNA levels with protein levels of different genes and (iv) mRNA levels with protein levels of same genes. This helps to obtain global insights into the stability and variability of co-expression and correlation of mRNA and protein levels.

**Results:**

Analysis of the simultaneous co-expression of mRNAs and proteins yields mainly weak correlations. Therefore we introduce the concept of time-delayed co-expression patterns. Based on a time-course dataset, we obtain a high fraction of time-delayed correlations. In group (i), 67% of different transcripts are significantly correlated. At the protein level (ii), 68% of different proteins are significantly correlated. Comparison of the different molecular levels results in a 74% fraction of correlated transcript and protein levels of different genes (iii) and 56% for the same genes (iv). Furthermore, a higher fraction of protein levels (simultaneously 20% and short time-delayed 29%) is correlated than at the transcript level (10% and 18% respectively). Analysis of the dynamics of the correlation shows that correlation at the transcript level is largely passed to the protein level. In contrast, specific co-expression patterns are changed in multiple ways.

**Conclusions:**

Our analysis reveals that the regulation of transcription and translation contains a time-delayed component. The correlation at the protein level is more synchronous or delayed by shorter time than those at the transcript level. This supports the hypothesis that a higher degree of direct physical interactions require a higher synchronicity between the interacting partners. The conservation of correlation between the transcript level (i) and the protein level (ii) sheds light on the processes underlying transcription, translation and regulation. A future investigation of the conditions of conservation will give comprehensive insights in the complexity of the regulatory mechanisms.

## Background

Expression of genes can be divided into the two fundamental stages of mRNA and protein expression, both stages are regulated by complex mechanisms. Both mRNAs and proteins as dynamic and active macromolecules fulfil specific functions just like citizens in a society. They interact with each other to maintain the order, activity and stability of the cellular society.

Currently, most genome-wide expression studies focus on identifying coordinated genes at the mRNA level, extracting gene clusters and inferring gene regulatory networks [1-5]. Furthermore, similarity between mRNA profiles has been widely used to infer the cellular phenotype state for identification and detection of cancer subtypes [1,6-8]. In another study, mRNA co-expression has been systematically investigated in several eukaryotic species to detect hierarchical patterns represented as trees. These patterns recur in different pathways and exhibit linear, nonlinear, local, global, time-delayed, monotonic and non-monotonic characteristics. They reflect cellular inner regulation of mRNA levels and enhance our understanding of gene expression activity in the cellular society [9]. For the analysis at the protein level, two-dimensional gel electrophoresis coupled with mass spectrometry (2DE-MS) has been widely used to generate protein expression profiles [10,11]. Based on such data, many researchers have analyzed the correlation between mRNA and protein expression of the same gene [12-16]. However, no comprehensive study has been performed to decipher global interaction and mutual regulation among mRNAs and proteins. This is surprising since mRNA expression depends on protein expression while protein expression is based on mRNA expression. Most mRNA expression is directly regulated by proteins bound to DNA or indirectly controlled by complex protein-protein interactions (PPI), for example as co-factors. The reverse link from mRNA to proteins is manifested in the translation process. As a result, mRNA and protein levels in a cell should be correlated to a certain degree. Correlation between mRNA levels will not necessarily be conserved in the corresponding protein levels because of differences in post-transcriptional modifications and regulations. The impact of these mechanisms on the propagation of co-expression from mRNA to protein levels has not yet been systematically investigated. To prevent an overestimation of disrupted propagation, the dynamics of regulatory processes need to be incorporated. The dynamics introduce a time delay since the corresponding molecular processes are not instantaneous.

In this article, we perform a large-scale analysis of mRNA-mRNA, protein-protein and mRNA-protein co-expression. Based on two eukaryotic species, *Plasmodium falciparum *and human, we calculate correlation patterns between different genes and for mRNA-protein co-expression also for the same genes. We employ the high-throughput mRNA and protein expression profiles to obtain significance values for the correlation. This allows a statistically solid analysis of the correlation patterns and their propagation from mRNA to protein levels. This is an advance to the systematic understanding of the regulation of gene transcription and translation in a cellular society.

## Results

The data comprise mRNA transcript and protein sets of two different eukaryotic species: *Plasmodium falciparum*(*Pfa*) [13] and human [17,18] (for details see Table 1).

**Table 1:** Data summary.

Species	Data type	mRNAs	Proteins	Samples
*Pfa*	Life cycle	2502	2502	6

human	NCI60	86	86	59

The transcriptome and proteome data of the *Plasmodium falciparum *dataset are taken from a pioneering study from Le Roch KG et al [13]. In this study, the abundance of mRNA transcripts is calculated by applying the MOID algorithm for high-density oligonucleotide array analysis. The MOID algorithm provides a p-value for each measurement and thus a metric to evaluate the confidence of each data point. Transcripts are considered to be present if their expression levels are greater than 10 and the log of the p-value (logP) is less than -0.5. Applying this methodology, 4292 transcripts are detected in at least one of the six stages examined. On the protein level, point were measured using the Redi Micro BCA protein assay system (Pierce), 2904 proteins are detected in at least one of the seven stages. There are 2584 genes, which have a transcript and a protein in at least one stage. We discard 82 genes, which have a single transcript in the additional stage at the transcript level, arriving at 2502 genes for our analysis. These genes have a transcript and a protein in at least one of the six common stages (Mero, Ring, Troph, Schiz, Gameto and Sporo). In the human dataset, the abundance of mRNA transcripts of 60 human cancer/tumor tissues are tested with the Affymatrix U95 chip. The intensities of the probes in each probe-set are combined and normalized using GCRMA [17]. Proteomic profiling of the NCI-60 cancer cell lines is performed by new high-density reverse-phase lysate microarrays (RPLA). For 176 antibodies, signal intensities are measured and processed by the DI25 algorithm (log2) [18]. We compare transcript and protein expression profiles across the NCI60 cancer cell panel, which is based on nine tissues of origin. This results in 86 genes, which have both mRNA and protein expression levels available.

We analyze four different groups of correlation: (i) transcript levels of different genes, (ii) protein levels of different genes, (iii) mRNA levels with protein levels of different (iv) and same genes. For the first three groups (i) to (iii), we randomly sample gene pairs to investigate the co-expression. For the fourth group (iv), we use the same gene for transcript and protein levels.

### Discovering simultaneous co-expression across different molecular levels

We select gene pairs with statistically significant *γ *values under hypothesis testing procedure *T *(see Methods). The complete comparison of the correlations within each molecular level is shown in Table 2. Only a small proportion of gene pairs is correlated if one neglects a time delay. We call this simultaneous co-expression. In general, there are more gene pairs with significant correlation at the protein level than at the transcript level. As direct physical interaction is more important for proteins than for mRNAs, the results confirm the expectation that proteins accordingly require expression in a more coordinated fashion.

**Table 2:** Simultaneous co-expression within different molecular levels.

Species	Pairs	Correlated	No relationship
*Pfa*	mRNA-mRNA	2623(10.48%)	22397(89.52%)
	Protein-Protein	5016(20.05%)	20004(79.95%)

human	mRNA-mRNA	499(13.65%)	3156(86.35%)
	Protein-Protein	534(14.61%)	3121(85.39%)

Proteins bound to DNA regulate either directly and/or indirectly with mRNAs. Since these proteins interact with other proteins, mRNAs are also affected by protein-protein interactions (PPI). Reversely, translation connects mRNA levels with protein levels. Assuming only a weak impact of post-transcriptional regulation and time delays, this would increase the correlation between the transcript and the protein levels. Here, we investigate the correlations between the transcript and protein level based on different and identical genes. Afterwards we relax our assumption about the weak impact of time delays.

Table 3 shows the results for simultaneous co-expression for mRNA-protein pairs. There is a considerable proportion of correlated mRNA-protein pairs in each comparison. For both organisms, the correlation between the transcript and the protein expression of the same gene (iv) is higher than for different genes (iii). For *Plasmodium falciparum*, the difference is only about 17% while for human almost 41% of group (iv) is correlated in contrast to 6% of group (iii). The simultaneous co-expression is mainly driven by the translation of mRNA to proteins. Since the mRNA is taken as input while the protein is the product of the translation, correlation between these two levels for group (iv) is not surprising.

**Table 3:** Simultaneous co-expression between mRNA and protein level.

Species	Pairs	Correlated	No relationship
*Pfa*	mRNA-Protein (different genes)	7708(15.40%)	42332 (84.60%)
	mRNA-Protein (same gene)	432(17.27%)	2070(82.73%)

human	mRNA-Protein (different genes)	10(5.81%)	162(94.19%)
	mRNA-Protein (same gene)	35(40.70%)	51(59.30%)

### Discovering time-delayed gene co-expression across different molecular levels

According to the above analysis, only small proportions of gene pairs exhibit significant co-expression except for the human group (iv). This might be due to the assumption that time delay effects do not play an important role. In the cell, mRNAs and proteins interact in a complex molecular network, as time-dependent dynamics are intrinsic to such interactions, time delayed patterns between molecules should widely occur in the cellular context. We incorporate the time delay effect by calculating correlation between different time points. For the *Plasmodium falciparum *dataset, the mRNA and protein levels are detected in six life cycle stages. Ensuring sufficient data points to calculate the correlation, we use a maximal time delay of three time points. For larger delays, the expression vectors are too small such that the correlation becomes erratic. Hence, we investigate co-expression for simultaneous, delay of one, delay of two and delay of three time points.

Table 4 shows there are 66.64% (17.63 + 17.99 + 31.02) mRNA-mRNA pairs, 68.11% (29.35 + 22.41 + 16.35) protein-protein pairs, 73.57% (28.28 + 24.78 + 20.51) mRNA-protein pairs for different genes and 56.29% (17.31 + 17.19 + 21.79) mRNA-protein pairs for the same genes, which have time-delayed co-expression patterns. Comparing with the simultaneous co-expression proportion, it is clear that the time delay effect dominates the *Plasmodium falciparum *dataset. Confirming our previous results, the co-expression between different genes at the protein level is stronger than at the transcript level. There are only 12% of the gene pairs, which do not exhibit any correlation at the protein level in *Plasmodium falciparum *dataset. Moreover, co-expression at the same time point and, delayed by one or two time points among different genes at the protein level are persistently larger than those at the transcript level. This further indicates that genes at the protein level are more synchronized than at the mRNA level. Only the number of three time-point delayed co-expression at the transcript level is exceptionally high. Since co-expression is transferred between the protein and the transcript level, this high fraction of co-expressed genes might be due to an accumulation of synchronizing mechanisms at different levels. The mRNA-protein pairs among different genes as well as among same genes represent indirect interactions between molecules. The large number of time-delayed correlation between these pairs provides evidence that indirect molecular interaction in cellular process mainly behave by time-delayed coordination.

**Table 4:** Time-delayed co-expression.

Species	Pairs	simultaneous	One time- point delayed	Two time- point delayed	Three time- point delayed	no relationship	*p *Value
*Pfa*	mRNA-mRNA	2623 (10.48%)	4410 (17.63%)	4501 (17.99%)	7762 (31.02%)	5724 (22.88%)	*χ*^2 ^= 3617.32 *p *< 0.05
	protein-protein	5016 (20.05%)	7344 (29.35%)	5606 (22.41%)	4092 (16.35%)	2962 (11.84%)	
	mRNA-protein (different genes)	7708 (15.40%)	14150 (28.28%)	12399 (24.78%)	10265 (20.51%)	5518 (11.03%)	*χ*^2 ^= 639.42 *p *< 0.05
	mRNA-protein (same genes)	432 (17.27%)	433 (17.31%)	430 (17.19%)	545 (21.79%)	662 (26.46%)	

### Discovering positive regulation mechanisms across different molecular levels

**Figure 1:**
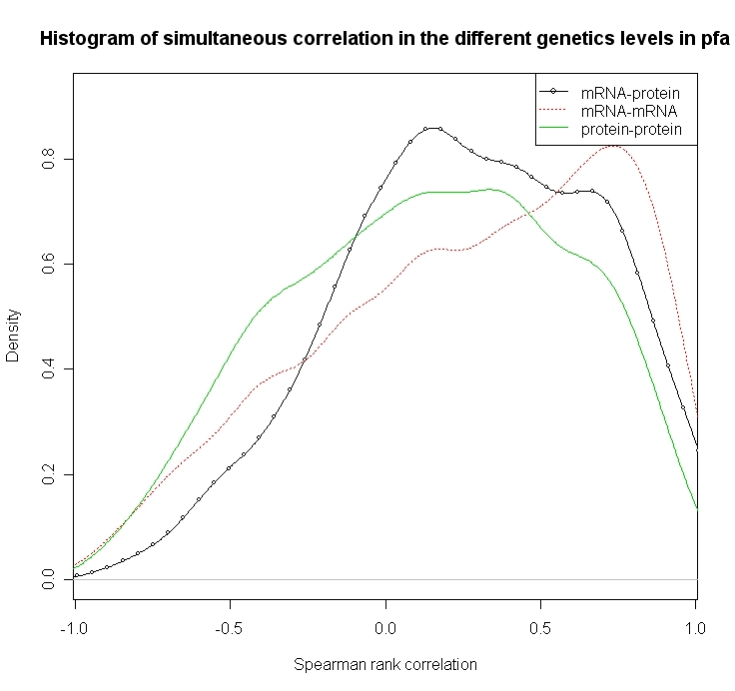
**Distribution of Spearman correlation values *r *for simultaneous co-expression values for different genes pairs within the same molecular level (mRNA-mRNA and protein-protein) as well as between the different molecular levels (mRNA-protein) in *Plasmodium falciparum***.

**Figure 2:**
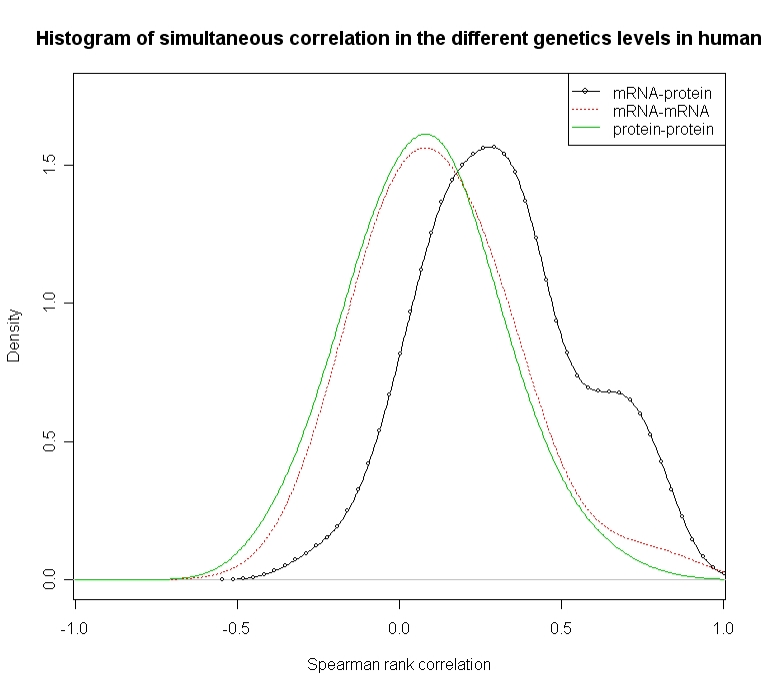
**Distribution of Spearman correlation values *r *for simultaneous co-expression values for different genes pairs within the same molecular level (mRNA-mRNA and protein-protein) as well as between the different molecular levels (mRNA-protein) in human**.

**Figure 3:**
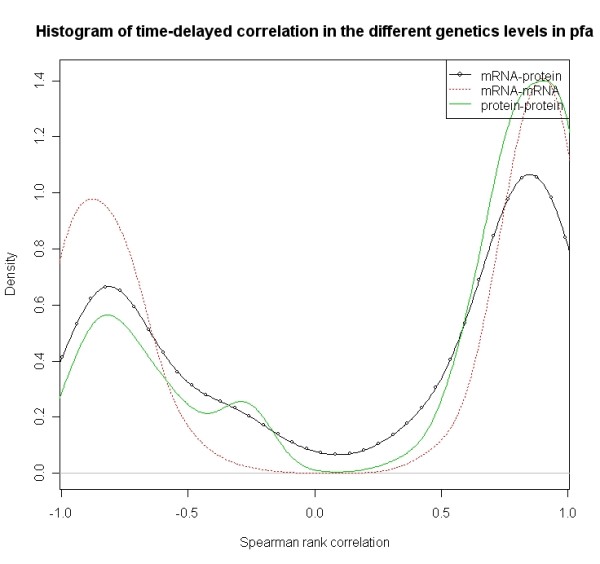
**Distribution of Spearman correlation values *r *for time delayed co-expression values for different genes pairs within the same molecular level (mRNA-mRNA and protein-protein) as well as between the different molecular levels (mRNA-protein) in *Plasmodium falciparum***.

Figure 1, Figure 2 and Figure 3 respectively show the distributions of simultaneous and time-delayed Spearman rank correlation values in *Plasmodium falciparum *and human. The correlation values for simultaneous co-expression (Figure 1) are prone to a positive correlation. This supports the hypothesis that genes are inclined to positive regulatory mechanisms. The similar phenomenon also appears in human (Figure 2). Interestingly, the time-delayed correlation values (Figure 3) follow a bimodal distribution with symmetry at zero. Despite the symmetry, the correlation values are also prone to positive correlation.

### Gene correlation transfer from transcript level to protein level

**Figure 4:**
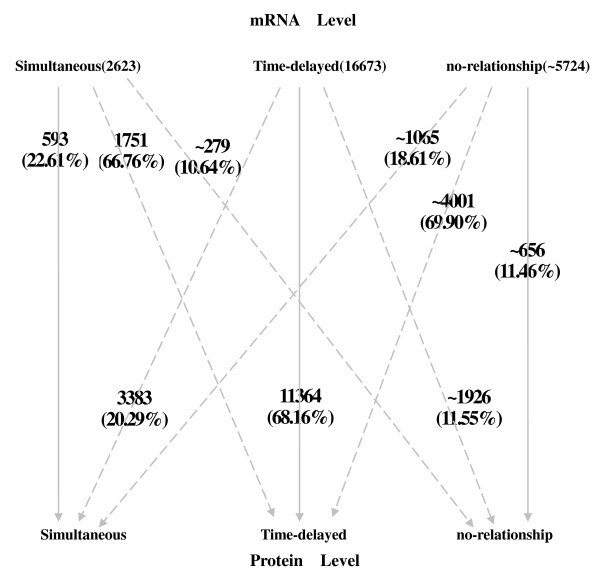
**Co-expression transfer of gene pairs from mRNA level to protein level in *Plasmodium falciparum***.

Figure 4 shows how the co-expression is transferred from transcript level to protein level in *Plasmodium falciparum*. Each gene pair is assigned to one of the three groups: simultaneous co-expression, time-delayed co-expression and no relationship. Based on this assignment, we track each gene pair from the transcript level to the protein level. The solid lines in Figure 4 denote gene pairs, which are assigned to the same groups at transcript level and protein level. These pairs have conserved co-expression across the molecular levels. Dashed lines indicate a change of the group. For example, the majority of simultaneous co-expressed gene pairs at transcript level are assigned to time-delayed co-expression at protein levels. This supports the hypothesis of an accumulation of time-delayed co-expression also at the transcript level.

In more detail, 2623 gene pairs belong to the group of simultaneous co-expression at transcript level, 22.61% of these pairs stay in this group at protein level. Another 66.76% of the gene pairs change to time-delayed co-expression while the remaining gene pairs (10.64%) don't exhibit any correlation at the protein level. This means that simultaneous co-expression at transcript level is mostly transferred to the protein level but is subject to a time delay. The majority of all gene pairs (16673) have significant time-delayed co-expression at the transcript level. Most of these pairs (68.16%) also exhibit a time-delayed co-expression at the protein level. Some of these gene pairs have changed in the extent of the delay, which is not shown in the figure. A fraction of 20.29% of time-delayed gene pairs at transcript level turns into simultaneous co-expression at protein level. Only a small proportion of pairs (11.55%) don't show any co-expression at protein level. In summary, this indicates that time-delayed co-expression at transcript level is mainly transferred to the protein level with a smaller fraction inclining towards simultaneous co-expression. In comparison to simultaneous co-expression at transcript level, time-delayed co-expression is more stable underlying the importance of the temporal component of interactions. In addition, 5724 gene pairs don't have any measurable co-expression at transcript level. For the huge minority of those (69.90%), we detect time-delayed correlation at the protein level. Only 18.61% turn into simultaneous co-expression and 11.46% still have no relationship.

**Figure 5:**
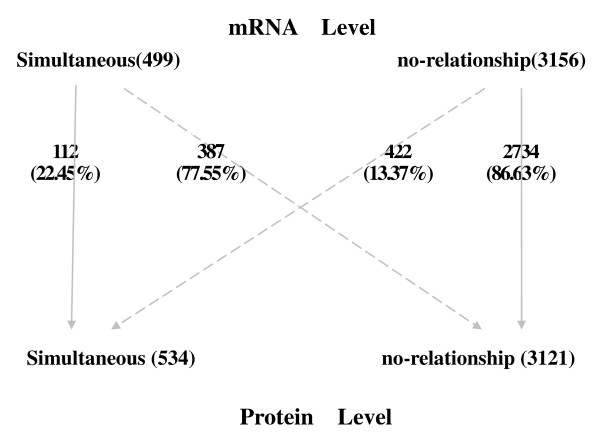
**Co-expression transfer of gene pairs from mRNA level to protein level in human**.

The human NCI60 dataset contains a series of cancer tissues including leukemia, melanoma, and cancer of ovarian, renal, breast, colon, lung, and central nervous system origin. Therefore, we cannot measure time-delayed co-expression. Hence, we restrict the analysis to the two groups of simultaneous co-expression and gene pairs without co-expression. The results are shown in Figure 5. Only 22.45% of simultaneous co-expressed gene pairs at transcript level are also co-expressed at the protein level. This is not surprising since as indicated by the *Plasmodium falciparum *dataset, most co-expressions are time delayed. Since the time-delayed co-expressed gene pairs are among the non co-expressed gene pairs, one would expect this result.

### Significant function categories of co-expression transfer patterns

We further investigate the biological context for gene pairs with different co-expression transfer patterns. We employ the subtree 'Molecular Function' of the Gene Ontology (GO). The GO term enrichment analysis is performed with the tool GOEAST [19]. We select function categories with a p-value less than 0.01. There are 83 terms enriched in human and three terms in *Plasmodium falciparum *for gene pairs with simultaneous co-expression both at transcript level and at protein level. These GO terms have as a distinct characteristic that most of them are involved in molecular binding and binding-related activity. The top four categories in human are DNA insertion or deletion binding, protein binding, MutLablpha complex binding, mis-match repair complex binding. In *Plasmodium falciparum*, the three enriched terms include nucleic acid binding, damaged DNA binding and translation elongation factor activity. Different binding actions can be further generalized in GO as a selective, non-covalent, often stoichiometric, interaction of a molecule with one or more specific sites on another molecule, reflecting direct physical interplay between molecules. Thus, the found binding categories actually provide us the supporting evidence that simultaneous co-expression at protein level explicitly represent the inner act conducted by directly physical binding between molecules in cellular society.

For genes pairs with time-delayed co-expression in *Plasmodium falciparum*, there are 76 significant terms both at transcript level and protein level. Most of those represent biological activity mainly involving transcription regulator, transporter and catalysis of a biochemical reaction. Proteins for transporter activity enable the directed movement of substances (such as macromolecules, small molecules, ions) into, out of, within or between cells. Proteins for enzymes are indirectly associated with each other by a series of catalyzed reactions. Thus, a distinct time delay caused by molecular movement or reaction process actually exists among these proteins. This is reflected by the time-delayed co-expression at protein level.

## Discussion

Our results show a high fraction of gene pairs, which are co-expressed either simultaneously or with a time delay within and between transcript and protein levels. This indicates that co-expression is a universal phenomenon for mRNA and proteins in a cellular society. The majority of co-expressed gene pairs are not simultaneously co-expressed but are shifted in time. This holds for gene pairs at transcript level and protein level. Le Roch, et. al. [13] illuminate time-delayed regulatory mechanisms by a global analysis of transcript and protein levels across the *Plasmodium falciparum *life cycle. According to their research, the gametocyte transcriptome correlates best with the proteome of the following gamete stage. This time shift is also observed for other stages, the merozoite stage transcriptome is correlated best with the ring-stage proteome, whereas the ring-stage transcriptome correlates best with the trophozoite proteome. Lee, et. al. [20] use genome-wide location data to identify six regulatory network motifs: autoregulation, multicomponent loops, feedforward loops, single-input, multi-input, and regulator chain. They reveal the time order of different molecules in the motifs and indicate time-shifted co-expression within the same network motifs. Our results further suggest the gene regulatory conducts the time-delayed regulatory mechanism, and the regulatory effect at the level of mRNA stability and/or translation is time shifted.

In *Plasmodium falciparum*, the correlation analysis of different genes and same genes across different molecular levels show that 88.97%(100-11.03)and 73.54%(100-26.46)gene pairs are co-expressed (Table 4). This suggests a universal correlation between the transcriptome and proteome in *Plasmodium falciparum*.

The correlation is also conserved from the transcript level to the protein level. These results seem to contradict most previous analyses on correlation between the transcriptome and the proteome which only report minor correlation [12,21-23]. These analyses focus on simultaneous correlation by neglecting time-delayed correlation. In fact, our results partially confirm this claim. Based on our analysis of simultaneous co-expression in two species, only a few gene pairs in *Plasmodium falciparum *are co-expressed (see Table 3). This holds for the transcript level, the protein level and between both levels. Only the incorporation of a time delay in the correlation analysis leads to the detection of considerable co-expression. Hence, the time delay introduced by molecular processes needs to be acknowledged in co-expression analysis at the transcript and protein level.

The analysis of co-expression of mRNA-mRNA pairs, mRNA-protein pairs and protein-protein pairs shows that the distribution of simultaneous co-expression values is prone to positive values. Two distinct peaks characterize the distributions of the time-delayed co-expression values. The high peak is centered around +0.9 and the low peak is located around -0.7 (see Figure 3). This indicates genes prefer to regulate positively rather than negatively during the process of transcript regulation and post- transcriptional regulation.

Both species have more co-expressed gene pairs at the protein level than at the transcript level (see Table 2). Incorporating time delays shows that simultaneous and short time delayed co-expression is more abundant at protein levels than at the transcript levels (see Table 4). This suggests that post-transcriptional regulation tends to be synchronous due to more direct physical interactions leading to synchronized protein expression.

Co-expression between gene pairs is partially conserved between the transcript and the protein level. Especially co-expression at the protein level strongly depends on co-expression at transcript level (see Figure 4). 50.41% ((593 + 11364 + 656)/25020) of the gene pairs keep the same co-expression patterns across the different molecular levels. A high number of gene pairs with any type of co-expression at the transcript level (68.31% (593 + 1751 + 11364 + 3383)/25020) are also co-expressed at the protein level. Still, the transfer of gene pair co-expression across the molecular levels shows flexibility. More than a third of the gene pairs (49.59%) change their type of co-expression across transcript and protein levels. For example, simultaneous co-expression is often changed to time-delayed co-expression while time-delayed co-expression sometimes turns into simultaneous co-expression. In addition, non co-expressed gene pairs can become co-expressed. Thus, the transfer of co-expression from transcript level to protein level in *Plasmodium falciparum *is characterized by both conservation and flexibility. In summary, co-expression at the transcript level partially reflects co-expression at the protein level as well as co-expression at the protein level is partially driven by co-expression at the transcript level. Hence, the analysis of gene pair co-expression transfer between different molecular levels gives comprehensive insights and enhances understanding of the complexity of gene regulatory mechanisms.

## Conclusions

Our analysis shows that simultaneous co-expression only resembles a part of the types of co-expression in a cellular society. It is important to include time delays in the analysis. At least for *Plasmodium falciparum*, this is the dominant type of co-expression among different gene pairs at the same molecular level, different gene pairs at different molecular levels and same gene pairs at different molecular levels.

Furthermore, different gene pairs at the same molecular level are more frequently co-expressed simultaneously or with a short time delay at the protein level than at the transcript level. This is due to more direct physical interactions between proteins, which require concerted expression.

We analyzed the effect of transcriptional and translational processes by investigating the co-expression of mRNA-protein gene pairs at the different molecular levels. Different genes at transcript and protein level exhibit more time-delayed regulatory mechanisms while considering the same gene at both levels shows more simultaneous co-expression.

Mainly, co-expression of gene pairs at the transcript level is passed to the protein level. Though, specific types of co-expression are changed in multiple ways. Therefore, the transfer of co-expression across molecular levels can be described as a harmonious process of both conservation and flexibility.

## Methods

**Figure 6:**
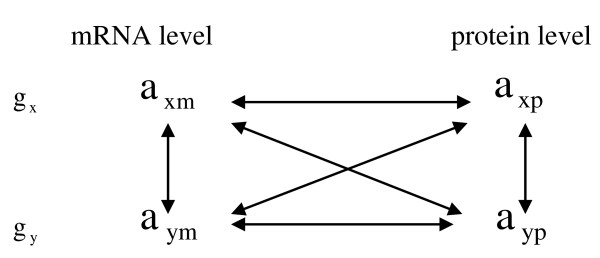
**Comparison strategy between different molecules**. *g*_*x *_and *g*_*y *_denote different genes, *a*_*xm *_and *a*_*xp *_denote the mRNA and protein expression intensities of gene *g*_*x*_. The connection between different molecules shows their correlations will be calculated.

We applied Spearman rank correlation *γ *to unravel co-expression between different genes and proteins. The Spearman rank correlation coefficient has been proposed for the comparison of transcriptome and proteome [16]. Furthermore, it is capable of capturing monotonic trends instead of only linear trends as Pearson correlation coefficient. As described below in more detail, we apply a hypothesis test *T *to determine the significance of a correlation based on a permutation approach. This method is more robust since it automatically takes into account tied data points. Figure 6 displays the comparison strategy between different molecules.

### Methods for unravelling simultaneous and time-delayed co-expression

The expression vectors of two genes, *g*_*x *_and *g*_*y*_, are denoted by *X *= (*x*_1_, *x*_2 _... *x*_*i *_... *x*_*N*_) and *Y *= (*y*_1_, *y*_2 _... *y*_*i *_... *y*_*N*_), with *x*_*i *_being the expression value of the *i*th experimental condition of *g*_*x*_, and correspondingly *y*_*i *_for the expression value of the *i*th experimental condition of *g*_*y*_.

The basic definition of the co-expression score *γ *for gene *g*_*x *_and *g*_*y *_follows from the Spearman rank correlation coefficient

with *L *denoting the dimension of *X *and *Y*. Here, we rank both *X *and *Y *from the highest to the lowest values. Then, we subtract the two sets of ranks to obtain the difference *d*.

If data are given as a time course experiment, time-delayed co-expression needs to be considered. Suppose gene *g*_*x *_is co-expressed with a time delay of *t *time points with respect to *g*_*y*_, we use truncated expression vectors *X *= (x_*t*+1 _... *x*_*i *_... *x*_*N*_) and *Y *= (*y*_1_, *y*_2 _... *y*_*i *_... *y*_*N*-*t*_) to calculate the correlation. Correspondingly, if *g*_*y *_is shifted by *t *time points, we employ the expression vectors *X *= (*x*_1_, *x*_2 _... *x*_*i *_... *x*_*N*-*t*_) and *Y *= (*y*_*t*+1 _... *y*_*i *_... *y*_*N*_). After calculating the score *γ *for each possible time shift, we predict a time delay of *t *time points by setting *t *to the time shift of the highest retrieved score *γ*, and the *p*-value is calculated and adjusted by multiple hypothesis testing to estimate the significance of score.

### Multiple hypothesis testing procedure *T*

We apply a hypothesis test for the co-expression score based on a permutation approach using Monte Carlo techniques. Based on this procedure, we can test whether a calculated score *γ *for two genes is a random sample from the background distribution of scores. The background distribution of scores is obtained by perturbing experimental conditions. For control of the overall false discovery rate, the *p*-value was further adjusted by the Bonferroni correction approach.

The test procedure is as follows:

(1) Create reference expression vectors of *g*_*x *_and *g*_*y *_under *H*_*0 *_by permuting experimental conditions of *X *and *Y*.

(2) Calculate co-expression score *γ*_*0 *_of permuted *X *and *Y*.

(3) Repeat step the two previous steps 500 times.

(4) Create cumulative distribution of *γ*_*0 *_(null distribution).

(5) Calculate *p*(γ | *H*_*0*_) after the Bonferroni correstion, if *p *< 0.05, reject *H*_*0*_.

Only gene pairs with significant co-expression scores are selected.

## Authors' contributions

HW conceived of this study, designed and performed the statistical analysis, drafted the manuscript. QW participated in design of the study and performed the biological analysis as well as revising the manuscript. UJP participated in discussing, revising and improving the manuscript. BS, JH, BW implemented the search for data and biological knowledge. XL participated in design and coordination. All authors read and approved the final manuscript.
